# FACE facts hold for multiple generations; Evidence from natural CO_2_ springs

**DOI:** 10.1111/gcb.14437

**Published:** 2018-11-13

**Authors:** Jasmine M. Saban, Mark A. Chapman, Gail Taylor

**Affiliations:** ^1^ Biological Sciences University of Southampton, Life Sciences Southampton UK; ^2^ Department of Plant Sciences University of California Davis California

**Keywords:** atmospheric CO_2_, climate change, meta‐analysis, natural CO_2_ spring, plant adaptation, plant response, plasticity

## Abstract

Rising atmospheric CO_2_ concentration is a key driver of enhanced global greening, thought to account for up to 70% of increased global vegetation in recent decades. CO_2_ fertilization effects have further profound implications for ecosystems, food security and biosphere‐atmosphere feedbacks. However, it is also possible that current trends will not continue, due to ecosystem level constraints and as plants acclimate to future CO_2_ concentrations. Future predictions of plant response to rising [CO_2_] are often validated using single‐generation short‐term FACE (Free Air CO_2_ Enrichment) experiments but whether this accurately represents vegetation response over decades is unclear. The role of transgenerational plasticity and adaptation in the multigenerational response has yet to be elucidated. Here, we propose that naturally occurring high CO_2_ springs provide a proxy to quantify the multigenerational and long‐term impacts of rising [CO_2_] in herbaceous and woody species respectively, such that plasticity, transgenerational effects and genetic adaptation can be quantified together in these systems. In this first meta‐analysis of responses to elevated [CO_2_] at natural CO_2_ springs, we show that the magnitude and direction of change in eight of nine functional plant traits are consistent between spring and FACE experiments. We found increased photosynthesis (49.8% in spring experiments, comparable to 32.1% in FACE experiments) and leaf starch (58.6% spring, 84.3% FACE), decreased stomatal conductance (g_s_, 27.2% spring, 21.1% FACE), leaf nitrogen content (6.3% spring, 13.3% FACE) and Specific Leaf Area (SLA, 9.7% spring, 6.0% FACE). These findings not only validate the use of these sites for studying multigenerational plant response to elevated [CO_2_], but additionally suggest that long‐term positive photosynthetic response to rising [CO_2_] are likely to continue as predicted by single‐generation exposure FACE experiments.

## Graphical abstract

Plant responses to future, elevated atmospheric CO_2_ from single‐generation FACE experiments were compared with those observed in naturally high carbon dioxide springs, following multi‐generation exposure. Responses for a panel of traits, including enhanced photosynthesis and reduced stomatal conductance, were broadly conserved suggesting that FACE experiments are a good proxy for long‐term, multi‐generational responses to this facet of the changing climate. These findings have implications for enhanced global greening, since our data suggest that vegetation will not acclimate to elevated, CO_2_ growth stimulation is likely to persist. Similarly, the data are relevant for future plant breeding and conservation strategies.

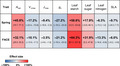

## 1 INTRODUCTION

Average atmospheric global [CO_2_] is now consistently above 400 ppm for the first time in around 23 million years of evolutionary time (Pearson & Palmer, 2000). Increased atmospheric [CO_2_] will be a key feature of future climates, and although there is clear resolve to cap atmospheric [CO_2_] to below 530 ppm in order to avoid catastrophic ecosystem change under global warming, it remains unclear whether these [CO_2_] targets will be met (Stocker, 2013). Despite the profound impact of [CO_2_] on plant functioning, future predictions of plant responses to elevated [CO_2_] are predominantly validated using experimental data derived from single‐generation experiments, which model only plant phenotypic plasticity. These plastic responses have been extensively quantified in experimental systems ranging from small controlled environment studies to large ecosystem experiments using FACE, and generalized through meta‐analyses that are used to inform or validate models and predictions (Ainsworth & Long, 2005; Dybzinski, Farrior, & Pacala, 2015; Vanuytrecht & Thorburn, 2017). While these experiments have played a pivotal role in informing short‐term projections of, for example, food security (Myers et al., 2014; Wheeler & Von Braun, 2013) and the likely distribution of plant ecotones in a changing climate (Barnaby & Ziska, 2012; Forkel et al., 2016; Smith et al., 2016), extrapolating to predict consequences of climate change for the end of the century may be precarious.

Beyond single‐generation plastic plant responses to elevated [CO_2_] there is some evidence for adaptation (the inheritance of derived characteristics that enhance fitness in a given environment) but a lack of conclusive evidence that elevated [CO_2_] could act as a selective agent on either genetic or epigenetic variation under climate change in the natural environment (Frenck, Linden, Mikkelsen, Brix, & Jørgensen, 2013; Leakey & Lau, 2012; Ward, Antonovics, Thomas, & Strain, 2000). Regardless, there is a wealth of evidence to suggest that transgenerational effects can and do contribute to plant response to elevated [CO_2_] over multiple generations (Jablonski, Wang, & Curtis, 2002; Johnston & Reekie, 2008; Springer & Ward, 2007).

Multigenerational experiments are a key challenge for the study of plant adaptation, owing to the time, energy and expense of growing plants under such conditions long‐term, especially for long‐lived and large plant species. Facilities are expensive and labour intensive to build and maintain, and cannot provide information on population responses to elevated [CO_2_] over generations in the timeframe needed to prepare for climate change. To this end, plants surrounding natural CO_2_ springs are a precious resource to further elucidate evolutionary adaptation and long‐term response to elevated [CO_2_]. Plants growing at natural CO_2_ springs have previously been utilized to study physiological response to rising [CO_2_] but have largely been abandoned due to concerns about CO_2_ emission variability over time and contamination by other exhaust gases. Here, we propose that as with other systems, provided these limitations are appropriately managed, spring sites represent a valuable resource that can contribute to our understanding of multigenerational plant response to elevated [CO_2_] in combination with other systems. In this first meta‐analysis of natural CO_2_ spring plant response to elevated [CO_2_], we highlight sites at which research has been conducted and synthesize available data, comparing responses to those in FACE experiments.

## 2 MATERIALS AND METHODS

### 2.1 Systematic search

To evaluate research at CO_2_ springs, we captured available data through a systematic search of the literature on 3rd July 2017. Using a structured string search and standard systematic review methodology, 3,294 studies were collated from Web of Science and screened according to strict inclusion criteria to provide a database of studies measuring traits in plants at natural CO_2_ springs compared to an ecologically similar control site in close proximity. These inclusion criteria are outlined in Supporting Information Appendix S1 and include (among others) that there must be a difference in [CO_2_] of at least 100 ppm between spring and control sites, and that sites are only included where contamination by [H_2_S] < 0.02 ppm and [SO_2_] < 0.015 ppm, as detailed in Supporting Information Table S1.

To avoid non‐independence as a result of multiple measurements of a trait being reported in a single publication, only one data point was taken for a trait for each species in each study. The data point extracted was decided on a trait by trait basis, for example photosynthetic measurements were taken at midday and during summer months if they were measured multiple times. In order to calculate effect sizes, mean, sample size and standard deviation were obtained from the text, tables or extracted from figures using DATATHIEF (Tummers, 2006). Authors were contacted if there was insufficient data reported for inclusion in the meta‐analysis and many authors kindly provided additional data.

Ultimately, we analysed data from 16 sufficiently replicated traits across 39 species in 25 papers (Supporting Information Appendix S2 and Table S1). This represents a subset of studies that have ever been used to study plant response at natural CO_2_ springs because we were unable to include traits (and therefore studies) where fewer than five species or studies measured the trait across the database.

### 2.2 Statistical analysis

#### 2.2.1 Effect size calculation

To compare trait differences between spring (elevated [CO_2_]) and control (ambient [CO_2_]) groups, we calculated the log response ratio (lnR) for each trait under elevated [CO_2_] as a metric for analysis. Log response ratio quantifies the proportional difference in population mean for a trait under elevated [CO_2_] at the spring site relative to ambient [CO_2_] at the control site. The log transformation is used to linearize the relationship between the two variables and to obtain residuals that are approximately symmetrically distributed where the sampling distribution may otherwise be skewed (particularly in small samples) (Hedges, Gurevitch, & Curtis, 1999). Log response ratio was calculated as:

$$\ln R=\ln\frac{{\overset{¯}{x}}_{\text{Spring}}}{{\overset{¯}{x}}_{\text{Control}}}=\ln\left({\overset{¯}{x}}_{\text{Spring}}\right)-\ln\left({\overset{¯}{x}}_{\text{Control}}\right)$$

where ${\overset{¯}{x}}_{\text{Spring}}$ is the mean trait value for plants growing under elevated [CO_2_] at the spring site and ${\overset{¯}{x}}_{\text{Control}}$ is the mean trait value for plants growing in ambient [CO_2_] at the control site. For more intuitive presentation, the log response ratio is converted to percentage difference using the formula [(*R*‐1) × 100]. All statistical analyses were performed in R version 3.2.2 (R‐CORE‐TEAM, 2015).

#### 2.2.2 Meta‐analysis

A random effects model was applied to calculate overall effect of elevated [CO_2_] on populations at the spring site relative to the control populations. Random effects models were used to account for environmental variation by assuming that true effect size varies between studies forming a distribution of effect sizes. The studies within the analysis are assumed to be a random sample of this distribution and the overall summary effect of a random effects model estimates the mean of the distribution of true effect sizes. The null hypothesis is that the mean of the distribution of effects is zero. The effect size of each species from each study was weighted using the inverse of its variance. All models used restricted maximum likelihood estimation. If a 95% confidence interval for a trait did not overlap zero then a significant response was considered in plants exposed to elevated [CO_2_] relative to their ambient counterparts at control sites.

#### 2.2.3 Assessing heterogeneity between studies

We examined variation between studies, partitioning it from within study error using the heterogeneity statistic *Q* and subsequently *I* ^2^ using the formula *I* ^2^ = 100% × (*Q*‐*df*)/*Q* (Higgins & Thompson, 2002). The *I* ^2^ statistic describes the percentage of variation across studies, that is due to heterogeneity rather than chance. Of the sixteen traits that were measured, the *Q* and *I* ^2^ statistics indicated that thirteen traits showed a significant degree of between‐study heterogeneity and effect sizes were calculated using a random effects model to account for this (Supporting Information Table S2). For three traits (*V*_cmax_, *J*_max_ and leaf carbon: nitrogen ratio), we found *Q* with *p* > 0.05 and/or an *I* ^2^ statistic <50% suggesting the variation in findings is compatible with chance alone (homogeneity) and therefore a fixed effect model was used to calculate these effect sizes.

Significant heterogeneity between studies existed for all traits analysed, suggesting that almost all of the variability in estimates was due to variation between samples rather than sampling error. This is common among ecological studies where an average *I* ^2^ of 83%–92% were reported in an analysis of ecological meta‐analyses (Senior et al., 2016). Given that individual samples come from a diverse array of global sites and from multiple functional groups, this heterogeneity is to be expected, but it is also useful to explore the basis of this heterogeneity by modelling potential moderator variables. Subgroup analysis was performed to examine trait changes in functional groups where sample size permitted (as trees, including both deciduous and evergreen trees, and herbs, including grasses, with forbs also analysed separately for stomatal conductance for comparison to FACE analyses), and a random effects meta‐regression model with defined moderator variables was fitted to the data to examine the effect of these moderator variables in the R package glmulti (Calcagno & De Mazancourt, 2010). Plant functional group and climate zone were used as moderator variables for meta‐regression analysis. For categorical variables, the category was considered an important predictor if the 95% confidence intervals of the category estimate did not overlap those of the overall effect size. Photosynthetic rate at growth [CO_2_] was the only trait where either of these categorical predictors were considered significant in predicting the estimate under meta‐regression. For this trait, we further decomposed the categorical variable “climate zone” to two continuous variables; average maximum daily temperature and annual precipitation for meta‐regression. Variance explained by a predictor variable was calculated through ANOVA of the model containing only this predictor variable versus the null model.

#### 2.2.4 Publication bias

In ecological studies, there may be a bias towards publishing positive and significant results, and studies with larger sample size have more power to detect significant differences, indeed Haworth, Hoshika, and Killi (2016) have suggested that publication bias has resulted in a significant over‐estimation of the impacts of elevated [CO_2_] on plants in FACE study meta‐analyses. Publication bias was quantified using weighted regression with multiplicative dispersion using standard error as the predictor to detect funnel plot asymmetry (the classical Egger's test), using the *regtest* function in the METAFOR package (Viechtbauer, 2010), by examining plots of the data and by estimating the fail‐safe number (Supporting Information Table S3; Rosenberg, 2005). From analyses of these tests and examination of the normal Q‐Q and funnel plots, we acknowledge that publication bias and the presence of outliers reduce confidence in the model estimates of summary effect for adaxial stomatal density, leaf chlorophyll content and leaf carbon content. Our interpretation of these results is duly cautious.

We additionally performed sensitivity analysis by applying weight functions to the effect sizes of studies to determine the impact of moderate publication bias. Assuming moderate selection of publication bias on the gathered dataset, we estimate that effect sizes in this study may be inflated by 6%–13%. This is similar in magnitude to the estimated inflation of FACE study effect sizes by 5%–15% due to moderate reporting bias (Haworth et al., 2016).

## 3 RESULTS

**Figure 1:**
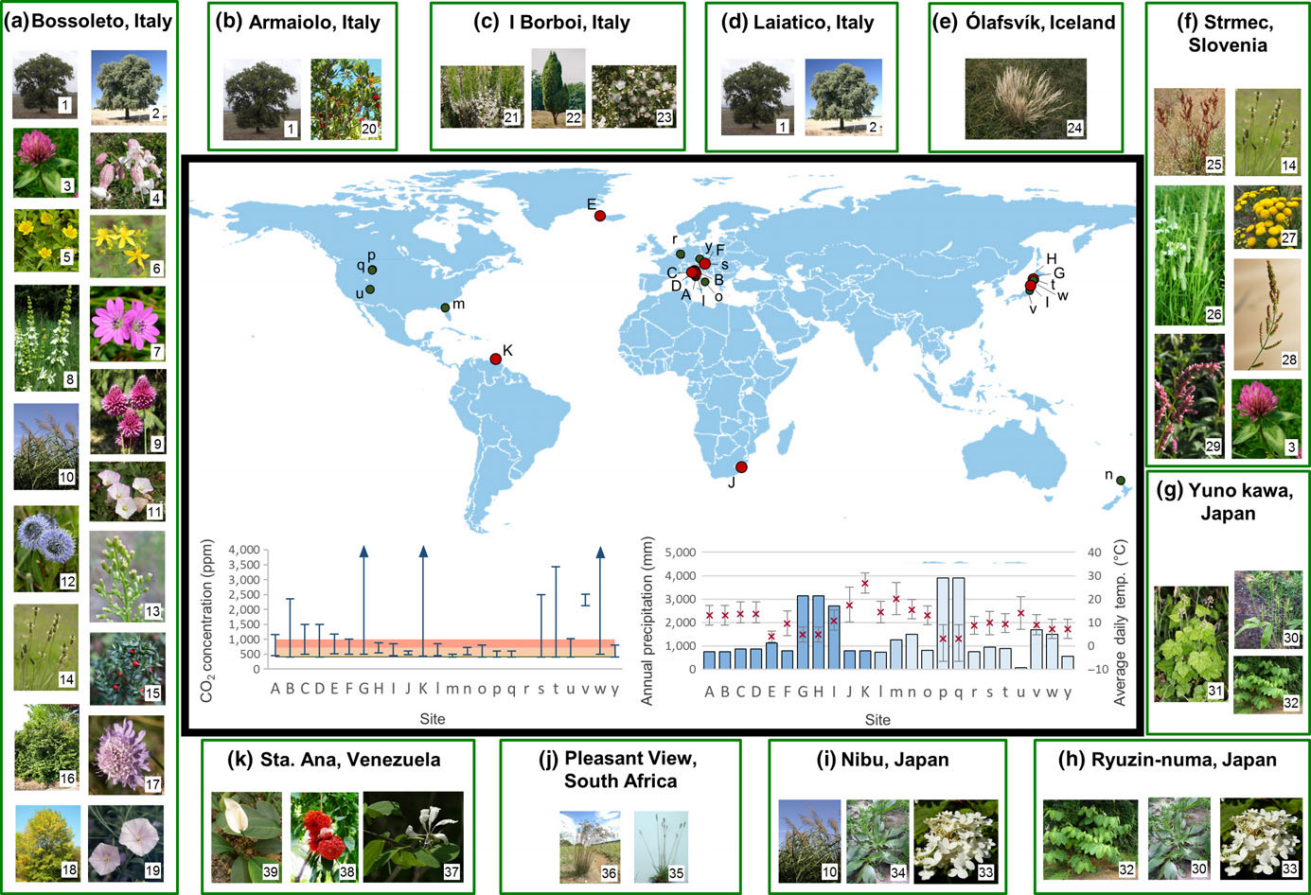
Sites of naturally elevated CO_2_ that have been used to study plant adaptation to elevated CO_2_. 24 sites are identified. Sites indicated by a red dot, and denoted with a capital letter were analysed in this meta‐analysis. Sites indicated by a green dot and denoted by lower case letters were not used by studies included in this meta‐analysis but studies at these sites have been published. Graphs show CO_2_ concentrations and climatic conditions of each site and the graph of CO_2_ concentration has predicted scenarios for the end of the century coloured from yellow to orange (IPCC, 2014). Green boxes for each of the sites used in the meta‐analysis show images of species represented in the meta‐analysis. Images were acquired from Google Images, labelled for reuse. Sites (l‐u) **l.** Solfatara, Italy, **m**. Ichetucknee springs, USA, **n**. Hakanoa springs, New Zealand, **o.** Orciatico, Italy, **p**. Ochre springs, USA, **q**. Mammoth upper terrace, USA, **r**. Laacher See, Germany, **s.** Rihtarovci, Slovenia, **t.** Tashiro, Japan, **u.** Burning hills, USA, **v.** Asahi, Japan, **w.** Kosaka, Japan, **y.** Plesná stream, Czech Republic. Species; 1. *Quercus pubescens*, 2. *Quercus ilex*, 3. *Trifolium pratense*, 4*. Silene vulgaris*, 5*. Potentilla reptans*, 6. *Hypericum perforatum*, 7. *Gerranium molle*, 8. *Stachys recta*, 9*. Allium sphaerocephalon*, 10*. Phragmites australis*, 11. *Convolvulus arvensis*, 12. *Globularia punctata*, 13. *Conyza candensis*, 14. *Plantago lanceolata*, 15*. Ruscus aculeates*, 16*. Buxus sempervirens*, 17*. Scabiosa columbaria*, 18. *Fraxinus ornus*,19. *Convolvulus cantabrica*, 20. *Arbutus unedo*, 21. *Erica arborea*, 22*. Juniperus communis*, 23. *Myrtus communis*, 24. *Nardus stricta*, 25. *Rumex crispus*, 26*. Phleum pratense*, 27. *Tanacetum vulgaris*, 28, *Echinochloa crus‐galli*, 29. *Polygonum hydropiper*, 30*. Sasa kurilensis*, 31. *Tiarella polyphylla*, 32*. Polygonum sachalinense*, 33*. Hydrangea paniculata*, 34*. Plantago asiatica*, 35. *Alloteropsis simialata,* 36. *Themeda triandra*, 37. *Bauhinia multinervia* 38. *Brownea coccinea*, 39*. Spathiphyllum cannifolium*

A systematic search of the literature revealed CO_2_ springs that have previously been utilized for this research occur extensively across the globe and range in latitude, temperature and rainfall (Figure 1). Significant differences in vegetation types and species present at each site are apparent, including many long‐lived tree species that are difficult to work with experimentally. The most comprehensively studied and characterized springs are located in Italy and Japan (Figure 1, Supporting Information Table S1)

**Figure 2:**
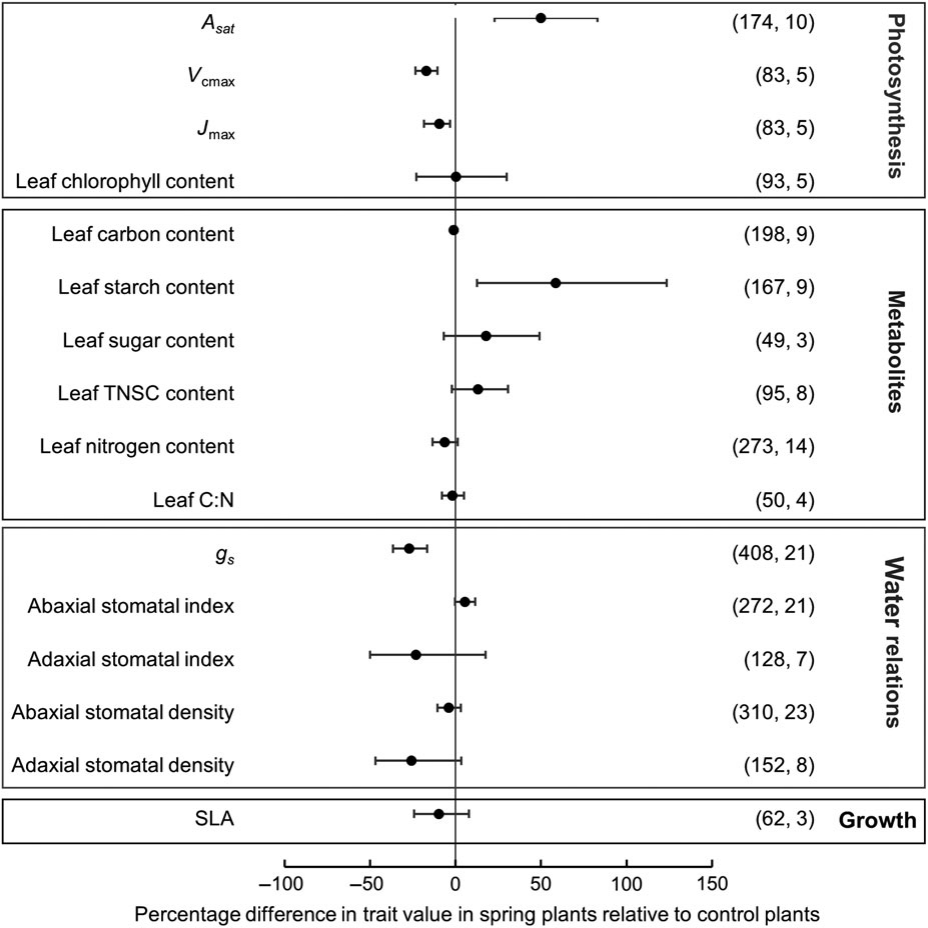
Trait percentage difference between plants at elevated and ambient [CO_2_] at naturally occurring CO_2_ springs and nearby control sites respectively; Meta‐analysis summary effect sizes. Traits: Photosynthetic rate at growth CO_2_ (A_sat_), maximum carboxylation rate (V_cmax_), maximum rate of electron transport (J_max_), leaf carbon content, leaf sugar content, leaf starch content, leaf total non‐structural carbohydrate (TNSC) content, leaf nitrogen content, leaf carbon to nitrogen ratio (C:N), stomatal conductance (*g*_s_), abaxial stomatal index ((stomatal density/(stomatal density +epidermal cell density)) × 100), adaxial stomatal index, abaxial stomatal density (stomata per unit area), adaxial stomatal density and specific leaf area (SLA). Symbols represent the percentage difference at elevated CO_2_ and their 95% confidence intervals. Total sample size (*n*) followed by the number of species included for each variable appear in parentheses after the symbol

**Figure 3:**
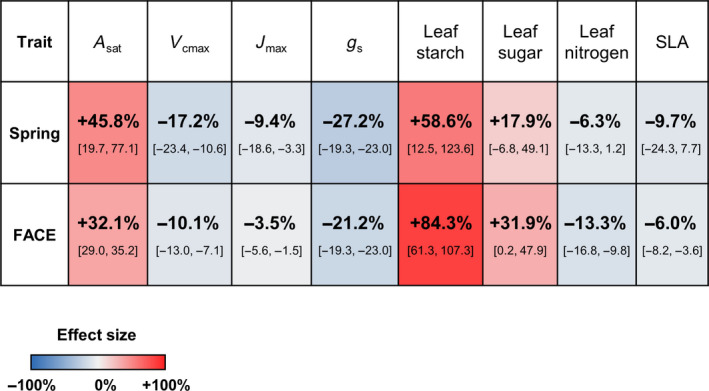
Comparison of long‐term response to elevated [CO_2_] in this CO_2_ spring meta‐analysis and short‐term response to elevated [CO_2_] in FACE meta‐analyses (Ainsworth & Long, 2005; Ainsworth & Rogers, 2007). Average percentage difference between plants growing at elevated versus ambient CO_2_ is given in bold with 95% confidence lower and upper boundaries given in square brackets. Squares are coloured according to the percentage difference as shown in the colour scale. Traits are photosynthetic rate at growth CO_2_ (*A*_sat_), maximum carboxylation rate (*V*_cmax_), maximum, rate of electron transport (*J*_max_), stomatal conductance (*g*_s_), leaf starch content, leaf sugar content, leaf nitrogen content, and specific leaf area (SLA)

Photosynthetic rate at growth [CO_2_] was significantly enhanced, by 49.8% (±10.6%) in spring versus control sites (Figure 2, Supporting Information Figure S1). This is comparable to the 31% enhancement observed in a meta‐analysis of plants at FACE facilities (Figure 3; Ainsworth & Long, 2005). Climate classification explained 60.9% of the variation in photosynthetic rate response to elevated [CO_2_], while functional group did not significantly explain variation. Much of the variation was attributable to studies at a site in Venezuela, where very high [CO_2_] was measured at the vents (27,000–35,000 ppm), and there was a lack of vertical characterization of [CO_2_] at the study site. This was also the only site in the tropical biome, highlighting that plant responses to elevated [CO_2_] in the tropics is a clear gap in our understanding of plant responses to elevated [CO_2_] globally (Jones, Scullion, Ostle, Levy, & Gwynn‐Jones, 2014). Using meta‐regression, average yearly maximum temperature was identified as a key component of photosynthetic response to elevated [CO_2_]. On average each 1°C increase in average maximum daily temperature increased the effect of elevated [CO_2_] on photosynthetic rate by 4.8% over the range of temperatures measured, a finding that is well supported by existing research (Ainsworth & Long, 2005; Wang, Heckathorn, Wang, & Philpott, 2012). The impact of elevated [CO_2_] on maximum carboxylation rate (V_cmax_) and maximum rate of electron transport (*J*_max_) were measured in fewer studies than photosynthetic rate. Effect sizes were calculated at −17.3% (±4.0%) and −9.4% (±3.4%) in spring versus control, respectively (Figure 2). The greater reduction in V_cmax_ relative to J_max_ suggests that where acclimation of photosynthesis occurs in these plants it is likely through a reduction in ribulose bisphosphate carboxylase content or activity.

A large and significant increase in starch content +58.6% (±19.1%) indicates that excess photosynthate from enhanced photosynthesis is increasingly converted to starch for storage for spring‐grown plants in response to elevated [CO_2_]. Leaf total non‐structural carbohydrates (TNSC) were not significantly increased +13.1% (±7.6%), and neither was leaf sugar content +17.9% (±12.7%). Additionally, no difference was seen in total carbon content in the leaves of plants at natural CO_2_ springs but with publication bias in this trait reducing confidence in the estimated effect size −1.6% (±0.7%).

**Figure 4:**
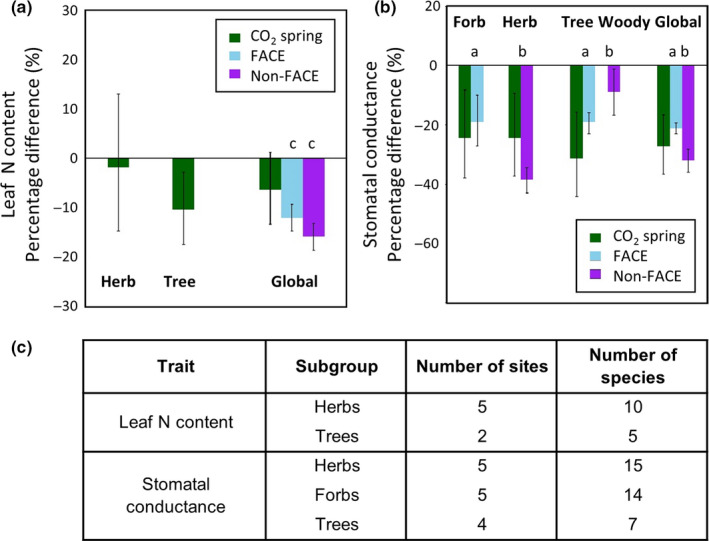
Percentage difference in (a) leaf nitrogen content and (b) stomatal conductance of plants growing at elevated relative to ambient [CO_2_]_._ Global effect size is presented, with subgroup analysis for this meta‐analysis of plants at CO_2_ springs (green), in comparison with a meta‐analysis of plants at FACE (blue) and non‐FACE (purple) facilities; a) Ainsworth & Long, 2005 b) Wang et al., 2012 and c) Loladze, 2014. c) The number of sites and species represented in subgroup analysis categories

When a global effect size was calculated, leaf nitrogen content did not differ between CO_2_ spring and control sites −6.3% (±4.0%), although the magnitude and direction of the effect size were consistent with those observed in FACE meta‐analyses. Spring sites typically have acidic soils (with pH 3.3–6.8, where recorded, at sites in this study, Supporting Information Table S1) and relatively anaerobic conditions which would predict higher soil concentrations of ammonium and reduced nitrate availability which could in part explain the apparent lack of photosynthetic acclimation seen in plants at CO_2_ springs (Bloom, Burger, Asensio, & Cousins, 2010; Onoda, Hirose, & Hikosaka, 2007). When functional groups were analysed separately in subgroup analysis, trees showed a significant decrease in leaf nitrogen −10.4% (±3.6%), while there was no significant difference in herbaceous plants or the global affect size across both functional groups (Figure 4). However, our estimation of leaf nitrogen content in trees was limited by the lack of replication across sites, with five species being measured in only two sites (Figure 4c).

A significant reduction in stomatal conductance of −27.2% (±7.2%) in plants at spring versus control plants suggests water savings through reduced transpiration, and this was of a similar magnitude to reduced stomatal conductance measured in FACE meta‐analysis (Figure 3, Supporting Information Figure S2). Although we acknowledge that our comparison to FACE and semi‐ or closed design (non‐FACE) meta‐analyses are confounded by differences in average CO_2_ concentration of studies (Table 1) this directional response is consistent across functional groups and experimental designs (Figure 4). There were no consistent responses in stomatal density (*SD*) or stomatal index (SI) to elevated [CO_2_] in springs (Figure 2). A decrease in *SD* may be observed more frequently for species exposed to elevated [CO_2_] in controlled environment (Woodward & Kelly, 1995) and FACE studies (Ainsworth & Rogers, 2007), with ~60% of studies in both analyses evidencing decreased *SD* under elevated [CO_2_]. In this meta‐analysis fewer than 50% of observations had decreased *SD* in spring sites, with average effect size of −4.0% (±3.7%), comparable to the non‐significant 5% decrease in FACE meta‐analysis (Ainsworth & Rogers, 2007).

**Table 1:** A comparison of the average CO_2_ concentration of experiments included in five meta‐analyses

Meta‐analysis	Experimental designs analysed	Average [CO_2_] of elevated treatments (ppm)
J. Saban, M.A. Chapman, and G. Taylor, (unpublished data)	Natural CO_2_ springs	791
Ainsworth and Long (2005	FACE	~560
Ainsworth and Rogers (2007	FACE	567
Wang et al. (2012	Semi‐open and closed systems	702
Loladze (2014	FACE	560
	Semi‐open and closed systems	732

Although SLA did not differ significantly between spring and control populations, the magnitude and direction of the effect size −9.7% (±9.41%) was consistent with FACE meta‐analyses (Figure 3). Since estimating increases in Leaf Area Index (LAI) to predict global greening and evapotranspiration under climate change depend upon changes in SLA, robust estimates of SLA response to elevated [CO_2_] based on empirical data is crucial to these predictions (De Kauwe et al., 2014). Meta‐analysis of SLA in plants at natural CO_2_ springs tends to support the decline in SLA in FACE meta‐analyses used to inform these models, but additionally suggests that some plant species may increase SLA under elevated [CO_2_]_,_ and this requires further investigation.

Across nine traits that had been measured in both this, the first meta‐analysis of response at spring sites, and comparable meta‐analyses of responses at FACE sites, eight traits were consistent in direction and magnitude (Figure 3). Leaf chlorophyll content was the only trait that was inconsistent in direction between the two meta‐analyses; however, the sample size of this trait for meta‐analysis at CO_2_ springs was small (with only five species studied) and was affected by publication bias. Other traits, such as leaf sugar content and SLA, although consistent in direction and magnitude showed larger variability than in FACE meta‐analyses. Whether this is solely an artefact of our small sample size compared to the large data availability for FACE meta‐analyses, or whether this is a result of comparing wild plants with the traditionally greater proportion of crop plants in FACE meta‐analyses is not discernible from this data set.

## 4 DISCUSSION

Here, we report the first meta‐analysis for data collected from plants in natural CO_2_ springs. Although these sites were initially suggested to study multigenerational plant response to elevated [CO_2_] in the early 1990s, this research was largely focussed on physiological and biochemical analysis since until recently, genomic technologies were unavailable for wild non‐model plant species such as those found at spring sites. We propose that they should now be re‐examined given the potential of new sequencing technologies to provide insight into future adaptive response to increased atmospheric [CO_2_]. Through meta‐analysis, we show that long‐term and multigenerational responses of plants to elevated [CO_2_] at natural CO_2_ springs are remarkably consistent with those measured in single‐generation FACE studies with eight of a panel of nine traits showing consistency. This is a key finding since it suggests that the magnitude and direction of long‐term response of plants to elevated [CO_2_] may be adequately predicted by single‐generation experiments, regardless of the mechanisms coordinating this response. The consequences of this finding may be wide‐ranging in supporting predictions of ecosystem change from models that have been parameterized with FACE data, for example the maintenance of positive photosynthetic rate which combined with other environmental factors may lead to the maintenance of global greening. Additionally, our results suggest that these sites are valuable to disentangle the role of transgenerational plasticity, adaptation and environmental constraints in the multigenerational response. This is particularly timely given the rapid recent progress in reduced cost of sequencing and software development for de novo genome and transcriptome assembly in non‐model organisms (Li & Harkess, 2018; Moreton, Izquierdo, & Emes, 2016).

A panel of eight traits in this study highlighted consistent response of FACE and spring‐grown plants. Altered gas exchange and photosynthetic rate are key features of the multigenerational response to elevated [CO_2_] and these trait differences were slightly enhanced relative to those at FACE sites (Ainsworth & Rogers, 2007). This may reflect the higher CO_2_ concentrations at spring study sites (800–1,000 ppm, representative of the “worst case” RCP8.5 climate scenario) relative to those across FACE sites (530–580 ppm, representative of the more moderate stabilization pathway RCP4.5) (IPCC, 2014) but suggests that photosynthetic rate is likely to be maintained despite environmental constraints and resource limitations, and over multiple generations. The magnitude of reduced stomatal conductance supports conclusions from FACE experiments that stomatal conductance does not acclimate to elevated [CO_2_] (Leakey et al., 2009) even over multiple generations, whether plastically coordinated or as the result of genetic assimilation or accommodation (Grossman and Rice, 2012). It is increasingly recognized that there is large variation in stomatal density (*SD*) response to elevated [CO_2_] both within and between species, and with significant dependence on other environmental factors (Haworth, Heath, & McElwain, 2010; Haworth, Killi, Materassi, & Raschi, 2015; Yan, Zhong, & Shangguan, 2017). In accordance with FACE meta‐analyses, our data provide no conclusive evidence that there is a general reduction in stomatal density in CO_2_ spring sites (Ainsworth & Rogers, 2007). Increased abaxial stomatal index was observed for some species but there was large variation across species, with a non‐significant mean effect size of 5.4% (±7.2%), which may indicate that decreases in *SD* result from expanding epidermal cells rather than a decline in stomatal initiation. Adaxial stomatal density and index were measured in fewer species and showed large variation. However, comparison between this meta‐analysis and the response of plants to elevated [CO_2_] in FACE experiments were limited because meta‐analyses of stomatal density (*SD*) response to elevated [CO_2_] in other systems (and many of the papers from which they take data) did not explicitly state whether *SD* was measured from the abaxial or adaxial leaf surface (Ainsworth & Rogers, 2007; Woodward & Kelly, 1995). Since the mechanisms of stomatal patterning on these surfaces are independent this is an important distinction, particularly because the ratio of stomata on these surfaces (and thus their role in gas exchange) is highly variable between species.

Although the sample size of this meta‐analysis was small, the study of plants growing in situ at natural CO_2_ springs meant that there was large diversity in plant species studied, which included functional groups such as trees that are difficult to study experimentally. Subgroup analysis of functional groups on traits evidenced that herbs growing at natural CO_2_ springs had enhanced photosynthetic rate, reduced stomatal conductance and no difference in nitrogen content of the leaves relative to control plants. Trees in contrast showed similarly enhanced photosynthetic rate and reduced stomatal conductance but a significant decrease in nitrogen content of the leaves at spring sites. These differences in leaf nitrogen content response between functional groups could be due to several factors not quantified here, including differences in nitrogen allocation, differential biotic interactions such as the association of mycorrhiza to trees versus herbs, or abiotic factors such as differential light availability or soil accessibility (Osada, Onoda, & Hikosaka, 2010; Ueda, Onoda, Kamiyama, & Hikosaka, 2017).

Interpretation of plant responses at CO_2_ springs would clearly be improved by further characterization of soil properties across the sites including nitrogen source (ammonium and nitrate availability), pH (characterized in just under half of sites globally) and soil CO_2_ concentration (Pfanz et al., 2007; Ueda et al., 2017). For example, there is limited information available on soil nitrogen at natural CO_2_ springs, but where quantified, total nitrogen pools have generally been found to be larger in spring than control soils (Newton, Bell, & Clark, 1996; Ross, Tate, Newton, Wilde, & Clark, 2000; Ueda et al., 2017). Of total soil nitrogen content, smaller inorganic nitrogen pools in CO_2_ spring sites may be indicative of increased uptake by plants under elevated [CO_2_] (Ueda et al., 2017), though nitrogen content of leaf litter returning to the soil generally shows decreased or unchanged nitrogen content at natural CO_2_ springs (Coûteaux, Kurz, Bottner, & Raschi, 1999, Cotrufo, Raschi, Lanini, & Ineson, 1999, Gahrooee, 1998, Ross, Tate, Newton, & Clark, 2002) suggesting changes in plant nitrogen allocation that may impact plant‐soil nitrogen cycling (see Gamage et al., 2018). Where investigated, and likely as a result of anaerobic and acidic soil conditions characteristic of natural CO_2_ springs, ammonium is the predominant form of inorganic nitrogen (Onoda et al., 2007; Osada et al., 2010; Ueda et al., 2017), which may facilitate the positive response of spring plant photosynthetic rate to elevated [CO_2_], since plants primarily utilizing ammonium as an inorganic nitrogen source will be less impacted by inhibition of nitrate assimilation by elevated [CO_2_] than plants utilizing nitrate (Bloom, 2015; Rubio‐Asensio & Bloom, 2016). Soil properties also influence the occurrence of soil microorganisms with impact on plant‐soil nutrient cycling which may well be key to understanding ecosystem response to long‐term CO_2_ exposure at natural CO_2_ springs. Microorganism populations including arbuscular mycorrhizal fungi (Maček, 2013; Maček et al., 2011; Maček, Kastelec, & Vodnik, 2012; Rillig, Hernandez, & Newton, 2000), archea (Krüger et al., 2011; Šibanc, Dumbrell, Mandić‐Mulec, & Maček, 2014), bacteria (Frerichs et al., 2013, Krüger et al., 2011, Šibanc et al., 2014, Videmšek et al., 2009), yeast (Šibanc et al., 2018), collembola (Hohberg et al., 2015) and nematodes (Hohberg et al., 2015; Pilz & Hohberg, 2015) show significant shifts in abundance and diversity at natural CO_2_ springs, especially towards acidophilic and anaerobic microorganisms (Krüger et al., 2011; Šibanc et al., 2014, 2018 ). This highlights the need for further characterization of soil properties and plant‐soil interactions at natural CO_2_ springs in order to interpret plant responses to elevated [CO_2_] at these sites and relate them to plant response to elevated [CO_2_] under climate change.

The potential for adaptation mediated by genetic change in plant populations exposed to elevate [CO_2_] is not well understood at present. Although genetic variation in traits responsive to elevated [CO_2_] has been evidenced in a wide range of plant taxa (De Costa, Weerakoon, Chinthaka, Herath, & Abeywardena, 2007; Lindroth, Roth, & Nordheim, 2001; Nakamura et al., 2011; Wieneke, Prati, Brandl, Stöcklin, & Auge, 2004; Ziska & Bunce, 2000) and this variation has been shown to be heritable in some studies (Case, Curtis, & Snow, 1998; Schmid, 1996), there remains significant debate over whether the strength of the elevated [CO_2_] signal is sufficient to induce an evolutionary response. Studies that have utilized reciprocal transplant or crossed factored experimental designs with natural populations of plants growing around CO_2_ springs have largely concluded that [CO_2_] can act as a selective agent because of significant differences in traits of spring and control plants when grown in ambient versus elevated [CO_2_] (Barnes et al., 1997, Nakamura et al., 2011, Onoda, Hirose, & Hikosaka, 2009, Polle, McKee, & Blaschke, 2001, Watson‐Lazowski et al., 2016), though this finding is not universal (Van Loon et al., 2016). A natural extension of research utilizing gradients and crossed factored experiments at natural CO_2_ springs is to combine this approach with High Throughput Sequencing (HTS) tools to further elucidate the role of adaptation and plasticity in the multigenerational response (Watson‐Lazowski et al., 2016). In addition epigenetic mechanisms have previously been highlighted as playing a role in coordinating plastic responses to elevated [CO_2_] (May et al., 2013) and the potential contribution of epigenetics to transgenerational plasticity under elevated [CO_2_] has not been explored, where natural CO_2_ springs can be combined with HTS tools such as methylation sequencing to provide insight.

The use of natural CO_2_ springs as a model for plant response to elevated [CO_2_] has largely fallen out of favour in the past two decades because of concerns about variability of gas emission over time and contamination with exhaust gases such as hydrogen sulphide (H_2_S) and sulphur dioxide (SO_2_). As a result, increasing emphasis on quantifying potential contaminants in sites that are actively used for research with the exclusion of those that do not meet requirements is evident in the literature (see Miglietta et al., 2012). In this meta‐analysis, we restricted the inclusion of data to springs with H_2_S contamination below thresholds that could affect plant functioning and those with recorded SO_2_ concentrations of below 0.015 ppm (Supporting Information Appendix S1). Although this threshold of [SO_2_] exceeds the minimal concentration expected to affect plant growth (0.01 ppm), it is less than concentrations recorded in and around industrialized cities globally (De Kok, Durenkamp, Yang, & Stulen, 2007). As with potential ethylene contamination of industrial CO_2_ in FACE sites, it is necessary to record and report these gas concentrations, both in initial site characterization and overtime to continually evaluate the suitability of the site as a model.

## 5 CONCLUSIONS

This first meta‐analysis of long‐term and multigenerational plant physiological responses to elevated [CO_2_] at natural CO_2_ springs has shown consistency in direction and magnitude with earlier observations in FACE, for eight traits related to gas exchange and physiology in a panel of nine traits. This suggests that predictions of plant response to rising [CO_2_] from single‐generation FACE studies are robust over multiple generations in short‐lived species and over long‐term exposure in long‐lived species, while highlighting that the role of ecological and evolutionary feedback in this response requires further investigation. This analysis supports the critical insights drawn from predictive models that incorporate empirical FACE data with relevance to food security, conservation and ecosystem change under climate change. Dissecting whether multigenerational responses are solely plastic, have an epigenetic basis, and/or if adaptive genetic accommodation or assimilation occurs, will require reciprocal transplant and crossed factored experiments (Nakamura et al., 2011; Watson‐Lazowski et al., 2016) which combined with newly accessible genomic technologies should provide crucial insight into the mechanistic basis of plant adaptation to elevated [CO_2_] in the near future. Nevertheless, our results suggest that single‐generation experiments have provided robust insight of wide‐ranging significance.
