# Supplementary material for: FACE facts hold for multiple generations; Evidence from natural CO2 springs
Source: Glob Chang Biol. 2018 Nov 13;25(1):1–11. doi: 10.1111/gcb.14437 (PMC7379517; doi:10.1111/gcb.14437)

SUPPLEMENTARY INFORMATION

Appendix S1 | Extended materials and methods information

Appendix S2 | Supplementary references

Supplementary Table S1 | Table of spring sites used to study plant responses to elevated CO_2

Supplementary Table S2 | Tests for heterogeneity in data collected for each trait measured across natural CO_2 spring sites

Supplementary Table S3 | Publication bias statistics for traits where publication bias was detected in CO_2 spring meta-analysis

Supplementary Figure S1 | Photosynthetic rate percentage difference between plants at elevated and ambient [CO_2] at naturally occurring CO_2 springs from individual studies included in this meta-analysis

Supplementary Figure S2 | Stomatal conductance (g_s) percentage difference between plants at elevated and ambient [CO_2] at naturally occurring CO_2 springs from individual studies included in this meta-analysis

Appendix S1: Extended materials and methods information

Search strings used for systematic search on 3rd July 2017 were as follows:

CO2 OR carbon dioxide) AND (spring OR natural OR enrichment/enriched OR mofette OR outgassing OR vent) AND (plant/s)

Systematic searches of the literature returned 3,294 studies which were screened for relevance by title, and relevant titles were then assessed by abstract for their potential to meet the following stringent inclusion criteria:

1) Publications must study naturally growing plant species at both a (terrestrial) spring site and a local control site with similar environmental conditions

2) There must be a minimum difference of 100 ppm in average daily CO2 concentration between designated spring and control sites

3) Control sites must have an average daily CO2 concentration below 435 ppm and spring sites must have an average daily CO2 concentration above 465 ppm

4) Traits measured must be quantitative for inclusion in the meta-analysis (for example studies only reporting presence/absence of species were not included)

5) At least three individuals must be sampled from each site per species, and at least two measurements must be made per plant (where the type of measurement taken allowed for this).

6) As required for effect size calculation, traits are only included if mean trait value, a measure of variance (standard error or standard deviation) and sample size are given in the study

7) Measurements of plants taken from springs with contamination by [H_2S] > 0.02 ppm or [SO_2] > 0.015ppm are not included in this analysis

Appendix S2: Supplementary references

Data provided to meta-analyses

1. Bettarini, I., Vaccari, F. P., & Miglietta, F. (1998). Elevated CO2 concentrations and stomatal density: observations from 17 plant species growing in a CO2 spring in central Italy. *Global Change Biology*, **4**, 17-22.
2. Blaschke, L., Schulte, M., Raschi, A., Slee, N., Rennenberg, H., & Polle, A. (2001). Photosynthesis, soluble and structural carbon compounds in two Mediterranean oak species (Quercus pubescens and Q. ilex) after lifetime growth at naturally elevated CO2 concentrations. *Plant Biology*, 3, 288-298.
3. Chaves, M. M., Pereira, J. S., Cerasoli, S., Clifton-Brown, J., Miglietta, F., & Raschi, A. (1995). Leaf metabolism during summer drought in Quercus ilex trees with lifetime exposure to elevated CO2. *Journal of Biogeography*, 255-259.
4. Körner, C., & Miglietta, F. (1994). Long term effects of naturally elevated CO 2 on mediterranean grassland and forest trees. *Oecologia*, **99**, 343-351.
5. Miglietta, F., Raschi, A., Körner, C., & Vaccari, F. P. (1998). Isotope discrimination and photosynthesis of vegetation growing in the Bossoleto CO2 spring. *Chemosphere*, **36**, 771-776.
6. Rapparini, F., Baraldi, R., Miglietta, F., & Loreto, F. (2004). Isoprenoid emission in trees of Quercus pubescens and Quercus ilex with lifetime exposure to naturally high CO2 environment. *Plant, Cell & Environment*, **27**, 381-391.
7. Scholefield, P. A., Doick, K. J., Herbert, B. M. J., Hewitt, C. S., Schnitzler, J. P., Pinelli, P., & Loreto, F. (2004). Impact of rising CO2 on emissions of volatile organic compounds: isoprene emission from Phragmites australis growing at elevated CO2 in a natural carbon dioxide spring. *Plant, Cell & Environment*, **27**, 393-401.
8. Schwanz, P., & Polle, A. (1998). Antioxidative systems, pigment and protein contents in leaves of adult Mediterranean oak species (Quercus pubescens and Q. ilex) with lifetime exposure to elevated CO 2. *The New Phytologist*, **140**, 411-423.
9. Stylinski, C. D., Oechel, W. C., Gamon, J. A., Tissue, D. T., Miglietta, F., & Raschi, A. (2000). Effects of lifelong [CO2] enrichment on carboxylation and light utilization of Quercus pubescens Willd. examined with gas exchange, biochemistry and optical techniques. *Plant, Cell & Environment*, **23**, 1353-1362.
10. Tognetti, R., Longobucco, A., Miglietta, F., & Raschi, A. (1998). Transpiration and stomatal behaviour of Quercus ilex plants during the summer in a Mediterranean carbon dioxide spring. *Plant, Cell & Environment*, **21**, 613-622.

11. Tognetti, R., Longobucco, A., Miglietta, F., & Raschi, A. (1999). Water relations, stomatal response and transpiration of Quercus pubescens trees during summer in a Mediterranean carbon dioxide spring. *Tree Physiology*, **19**, 261-270.
12. Tognetti, R., Johnson, J. D., Michelozzi, M., & Raschi, A. (1998). Response of foliar metabolism in mature trees of Quercus pubescens and Quercus ilex to long-term elevated CO2. *Environmental and Experimental Botany*, **39**, 233-245.
13. Tognetti, R., Minnocci, A., Peñuelas, J., Raschi, A., & Jones, M. B. (2000). Comparative field water relations of three Mediterranean shrub species co-occurring at a natural CO2 vent. *Journal of Experimental Botany*, **51**, 1135-1146.
14. Jones, M. B., Brown, J. C., Raschi, A., & Miglietta, F. (1995). The effects on Arbutus unedo L. of long-term exposure to elevated CO2. *Global Change Biology*, **1**, 295-302.
15. Peñuelas, J., Castells, E., Joffre, R., & Tognetti, R. (2002). Carbon-based secondary and structural compounds in Mediterranean shrubs growing near a natural CO2 spring. *Global Change Biology*, **8**, 281-288.
16. Peñuelas, J., Filella, I., & Tognetti, R. (2001). Leaf mineral concentrations of Erica arborea, Juniperus communis and Myrtus communis growing in the proximity of a natural CO2 spring. *Global Change Biology*, **7**, 291-301.
17. Tognetti, R., & Peñuelas, J. (2003). Nitrogen and carbon concentrations, and stable isotope ratios in Mediterranean shrubs growing in the proximity of a CO 2 spring. *Biologia Plantarum*, **46**, 411-418.
18. Cook, A. C., Tissue, D. T., Roberts, S. W., & Oechel, W. C. (1998). Effects of long-term elevated [CO2] from natural CO2 springs on Nardus stricta: photosynthesis, biochemistry, growth and phenology. *Plant, Cell & Environment*, **21**, 417-425.
19. Marchi, S., Tognetti, R., Vaccari, F. P., Lanini, M., Kaligarič, M., Miglietta, F., & Raschi, A. (2004). Physiological and morphological responses of grassland species to elevated atmospheric CO2 concentrations in FACE-systems and natural CO2 springs. *Functional plant biology*, **31**, 181-194
20. Pfanz, H., Vodnik, D., Wittmann, C., Aschan, G., Batic, F., Turk, B., & Macek, I. (2007). Photosynthetic performance (CO2-compensation point, carboxylation efficiency, and net photosynthesis) of timothy grass (Phleum pratense L.) is affected by elevated carbon dioxide in post-volcanic mofette areas. *Environmental and Experimental Botany*, **61**, 41-48.
21. Vodnik, D., Pfanz, H., Wittmann, C., Macek, I., Kastelec, D., Turk, B., & Batic, F. (2002). Photosynthetic acclimation in plants growing near a carbon dioxide spring. *PHYTON-HORN-*, **42**, 239-244.
22. Onoda, Y., Hirose, T., & Hikosaka, K. (2007). Effect of elevated CO 2 levels on leaf starch, nitrogen and photosynthesis of plants growing at three natural CO 2 springs in Japan. *Ecological research*, **22**, 475-484.
23. Stock, W. D., Ludwig, F., Morrow, C., Midgley, G. F., Wand, S. J., Allsopp, N., & Bell, T. L. (2005). Long-term effects of elevated atmospheric CO 2 on species composition and productivity of a

southern African C 4 dominated grassland in the vicinity of a CO 2 exhalation. *Plant Ecology*, **178**, 211-224.
24. Fernandez, M. D., Pieters, A., Donoso, C., Tezara, W., Azkue, M., Herrera, C., ... & Herrera, A. (1998). Effects of a natural source of very high CO 2 concentration on the leaf gas exchange, xylem water potential and stomatal characteristics of plants of Spatiphylum cannifolium and Bauhinia multinervia. *The New Phytologist*, **138**, 689-697.
25. Marín, O., Rengifo, E., Herrera, A., & Tezara, W. (2005). Seasonal changes in water relations, photosynthesis and leaf anatomy of two species growing along a natural CO2 gradient. *Interciencia*, **30**, 33-38.

Other sites that have been used to study plant response to elevated CO_2

Vejpustková, M., Thomalla, A., Cihak, T., Lomsky, B., & Pfanz, H. (2016). Growth of Populus tremula on CO2-enriched soil at a natural mofette site. *Dendrobiology*, *75*.
Krüger, M., Jones, D., Frerichs, J., Oppermann, B. I., West, J., Coombs, P., ... & Strutt, M. (2011). Effects of elevated CO2 concentrations on the vegetation and microbial populations at a terrestrial CO2 vent at Laacher See, Germany. *International Journal of Greenhouse Gas Control*, **5**, 1093-1098
Bartak, M., Raschi, A., & Tognetti, R. (1999). Photosynthetic characteristics of sun and shade leaves in the canopy of Arbutus unedo L. trees exposed to in situ long-term elevated CO 2. *Photosynthetica*, **37**, 1-16.
Miglietta, F., & Raschi, A. (1993). Studying the effect of elevated CO 2 in the open in a naturally enriched environment in Central Italy. *Vegetatio*, **104**, 391-400.
Onoda, Y., Hikosaka, K., & Hirose, T. (2005). Natural CO2 springs in Japan: A case study of vegetation dynamics. *Phyton*, **45**, 389-394.
Van Loon, M. P., Rietkerk, M., Dekker, S. C., Hikosaka, K., Ueda, M. U., & Anten, N. P. (2016). Plant–plant interactions mediate the plastic and genotypic response of Plantago asiatica to CO2: an experiment with plant populations from naturally high CO2 areas. *Annals of botany*, ***117***, 1197-1207.
Newton, P. C. D., Bell, C. C., & Clark, H. (1996). Carbon dioxide emissions from mineral springs in Northland and the potential of these sites for studying the effects of elevated carbon dioxide on pastures. *New Zealand Journal of Agricultural Research*, **39**, 33-40.
Ehleringer, J. R., Sandquist, D. R., & Philips, S. L. (1997). Burning coal seams in southern Utah: a natural system for studies of plant responses to elevated CO2. In *Plant Responses to Elevated CO2: Evidence from Natural Springs* (pp. 56-68). Cambridge University Press.
Woodward, F. I., Thompson, G. B., & McKee, I. F. (1991). The effects of elevated concentrations of carbon dioxide on individual plants, populations, communities and ecosystems. *Annals of Botany*, 23-38.

Sharma, S., & Williams, D. G. (2009). Carbon and oxygen isotope analysis of leaf biomass reveals contrasting photosynthetic responses to elevated CO 2 near geologic vents in Yellowstone National Park. *Biogeosciences*, **6**, 25-31.
Sajna, N., Meister, M., Bolhà r-Nordenkampf, H. R., & Kaligaric, M. (2013). Response of Semi-natural Wet Meadow to Natural Geogenic CO 2 Enrichment. *International Journal of Agriculture & Biology*, **15**.

Supplementary Table S1: Table of spring sites used to study plant responses to elevated CO_2

Country	Site	Data in meta-analysis?	Latitude	Longitude	Gas composition of at the spring study site			Soil pH		Koepper climate classification
					[CO_2]	[H_2S]	[SO_2]	Spring site	Control site	
**Sites used in this meta-analysis**										
Iceland	Ólafsvík	Y	64.9	-23.7	519-1179 ppmv	< 0.5 ppmv<br>No smell (< 0.025 ppmv)	-	-	-	Polar tundra (ET)
Italy	Armaiolo	Y	43.3	11.6	~500-2300 ppmmol	-	-	-	-	Temperate with dry hot summer (Csa)
	Bossoleto	Y	43.3	11.6	400-1200 ppmmol	0.022 ppmv	0.012 ppmv	5.6-6.8	7.5-7.7	Temperate with dry hot summer (Csa)
	I Borboi	Y	43.4	10.7	500-1500 ppmmol	0.060 ppmmol	0.004 ppmmol		6-7	Temperate with dry hot summer (Csa)
	Laiatico	Y	43.4	10.8	400-1500 ppmmol	0.022 ppmv	0.004 ppmv	6-7	6-7	Temperate with dry hot summer (Csa)
Slovenia	Strmec	Y	46.7	16	500-1000 ppmmol	-	-	5.0-5.2	5.0-5.2	Temperate without dry season, warm summer (Cfb)
Japan	Nibu	Y	38.5	140.0	~450-850 ppmmol	< 0.1 ppmv<br>No smell (< 0.025 ppmv)	-	3.6-4.2	4.1-4.3	Temperate without dry season, hot summer (Cfa)
	Ryuzin-numa	Y	40.7	141.0	~550-890 ppmmol	< 0.1 ppmv<br>No smell (< 0.025 ppmv)	-	3.5-3.7	2.8-3.4	Temperate without dry season, hot summer (Cfa)
	Yuno kawa	Y	40.7	141.0	~460-630 ppmmol	< 0.1 ppmv<br>No smell (< 0.025 ppmv)	-	3.7-4.5	3.7-4.5	Temperate without dry season, hot summer (Cfa)
South Africa	Pleasant View	Y	-30.7	30.02	~480-600 ppmmol	-	-	4.2-4.4	4.3-4.5	Temperate without dry season, warm summer (Cfb)
Venezuela	Sta. Ana	Y	10.6	-63.13	(S1) ~34200-35800 ppmmol at the vent<br>(S2) ~26800-27200 ppmmol at the vent	< 0.1 ppmmol	-	-	-	Tropical savannah (Aw)

					CO_2 concentrations approx. 1000					
**Other sites used to study plant response to elevated CO_2**										
Czech Republic	Plesná stream	N	50.1	12.46	>600 ppm at 50 cm vertical	-	-	-	-	Temperate without dry season, warm summer (Cfb)
Germany	Laacher See	N	50.4	7.25	Gradient 100-0% explored	-	-	4.0-6.0	5.5-6.3	Temperate oceanic climate (Cfb)
Italy	Orciatico	N	43.4	10.67	Avg. 465 ppmmol	-	-	-	-	Temperate with dry hot summer (Csa)
	Solfatara	N	42.5	12.13	450-850 ppmmol	0.245 ppmv	0.018ppmv	3.3-2.1	4.5-4.1	Temperate with dry hot summer (Csa)
Japan	Tashiro	N	40.7	140.92	400-1000 ppmmol	< 0.03 ppmmol	<0.03ppmmol	-	-	Temperate without dry season, hot summer (Cfa)
	Asahi	N	38.2	140	2123-2509 ppm	-	-	-	-	Temperate without dry season, hot summer (Cfa)
	Kosaka	N	40.4	140.8	503-7019 ppm	-	-	-	-	Temperate without dry season, hot summer (Cfa)
New Zealand	Hakanoa springs	N	-35.7	174.27	480-725 ppmv	< 0.18 ppmv	-	5.2-5.7	5.2-5.7	Temperate without dry season, warm summer (Cfb)
USA	Burning hills	N	37.3	-111.37	400-1000 ppm	-	-	-	-	Cold-desert climate (Bwk)
	Ichetucknee river springs	N	30.0	-82.76	450-500 ppmmol	-	-	-	-	Temperate without dry season, hot summer (Cfa)
	Ochre Springs	N	44.6	-110.4	419-482 ppmv	-	-	-	-	Dry summer subarctic (Dsc)
	Mammoth Upper Terrace	N	45.0	-110.7	401-607 ppmv	-	-	-	-	Warm-summer continental (Dfb)
Slovenia	Rihtarovci	N	46.6	16.1	Gradient 400 to >2500 ppmmol	-	-	-	-	Temperate without dry season, warm summer (Cfb)

Supplementary Table S2: Tests for heterogeneity in data collected for each trait measured across natural CO_2 spring sites

Statistic	Q stat	Df	p-val	I^2
Description	Is the variability in the observed effect size larger than would be expected based on sampling variability alone?			Percentage of total variation across studies that is due to heterogeneity rather than chance
Stomatal conductance	360.6	31	<0.0001	92.28%
Abaxial stomatal index	214.3	22	<0.0001	88.75%
Adaxial stomatal index	109.5	7	<0.0001	99.13%
Abaxial stomatal density	279.8	24	<0.0001	93.93%
Adaxial stomatal density	100.8	8	<0.0001	98.38%
Photosynthetic rate	180.7	8	<0.0001	96.47%
V_{cmax}	13.9	6	0.0312	56.42%
J_{max}	21.1	6	0.017	71.17%
Leaf chlorophyll content	34.8	7	<0.0001	80.88%
Leaf carbon content	63.3	14	<0.0001	78.69%
Leaf sugar content	172.5	4	<0.0001	95.94%
Leaf starch content	236.3	12	<0.0001	96.04%
Leaf total non-structural carbohydrate content	311.8	10	<0.0001	97.00%
Leaf nitrogen content	232.5	22	<0.0001	89.79%
Leaf carbon:nitrogen ratio	8.1	4	0.0893	49.87%
Specific leaf area	33.5	4	<0.0001	84.58%

Supplementary Table S3: Publication bias statistics for traits where publication bias was detected in CO_2 spring meta-analysis

**Trait**	**Egger's test for funnel plot asymmetry**		**Rosenthal's Fail-safe number**	
	**T test**	**P value**	**Fail-safe number**	**5N +10**
Abaxial stomatal index	2.7938	0.0109	92	125
Adaxial stomatal density	-3.1970	0.0151	95	55
Leaf chlorophyll content	2.5425	0.0439	0?	50
Leaf carbon content	-2.3206	0.0372	27	85

Supplementary Figure S1: Photosynthetic rate percentage difference between plants at elevated and ambient [CO_2] at naturally occurring CO_2 springs from individual studies included in this meta-analysis. Author(s) and species appear on the left hand side, numbers on the right hand side are effect size, with 95% confidence intervals in parentheses. Squares indicating mean effect size are drawn proportionally to the precision of the estimate. The summary polygon at the bottom of the plot indicates the mean effects size when all 20 estimates are analysed together using a random effects model. Note that in subgroup analysis plants were categorised as trees, which included both 'deciduous trees' and 'evergreen shrubs or trees', or herbs, which included 'herbaceous forbs' and grasses, rather than the functional groups used for visualisation here.

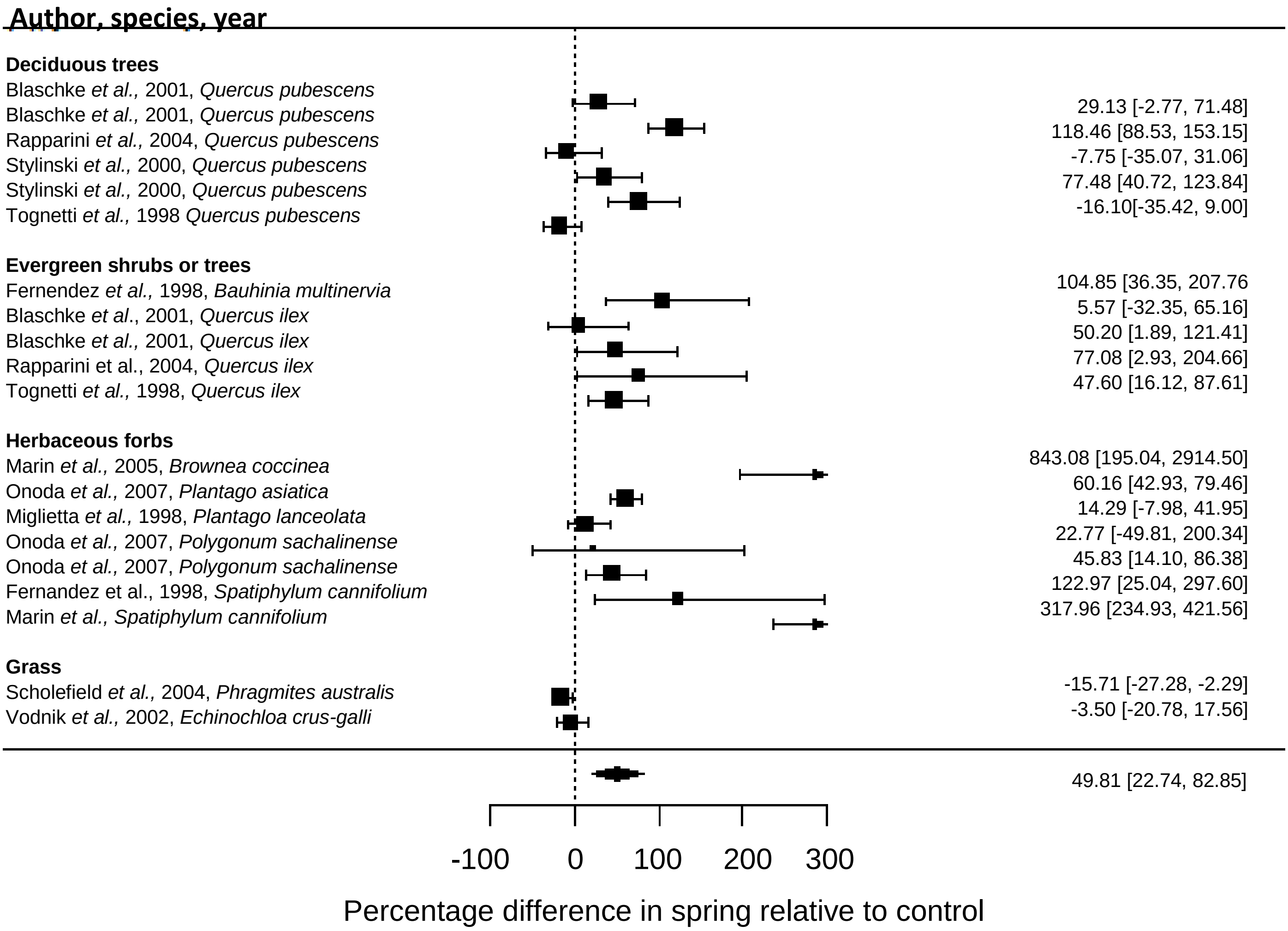

Supplementary Figure S2: Stomatal conductance (g_s) percentage difference between plants at elevated and ambient [CO_2] at naturally occurring CO_2 springs from individual studies included in this meta-analysis. Author(s) and species appear on the left hand side, numbers on the right hand side are effect size, with 95% confidence intervals in parentheses. Squares indicating mean effect size are drawn proportionally to the precision of the estimate. The summary polygon at the bottom of the plot indicates the mean effects size when all 32 estimates are analysed together using a random effects model. Note that in subgroup analysis plants were categorised as trees, which included both 'deciduous trees' and 'evergreen shrubs or trees' or herbs, which included 'herbaceous forbs' and grasses, or just herbaceous forbs, rather than the functional groups used for visualisation here.

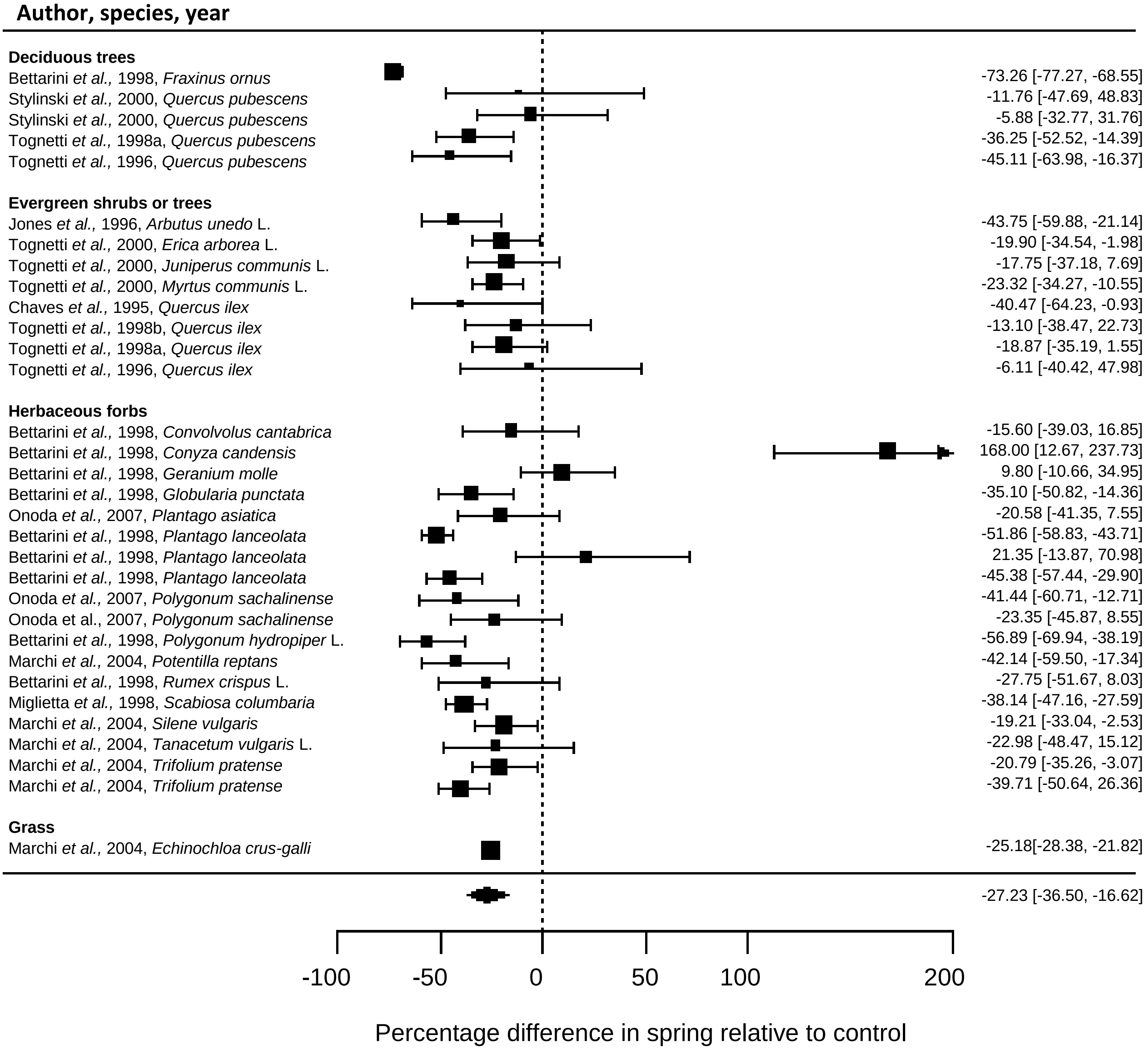

Supplement: Supplementary file 1 [file GCB-25-1-s001.pdf]
